# Ultrasonic Non-Destructive Testing and Evaluation of Stainless-Steel Resistance Spot Welding Based on Spiral C-Scan Technique

**DOI:** 10.3390/s24154771

**Published:** 2024-07-23

**Authors:** Liang Yang, Rongyan Chuai, Guixi Cai, Dan Xue, Jingming Li, Kunlin Liu, Chang Liu

**Affiliations:** 1 School of Information Science and Engineering, Shenyang University of Technology, Shenyang 110870, China; lyang@imr.ac.cn (L.Y.); me_sut@163.com (R.C.); x2d2003@163.com (D.X.); 2 Institute of Metal Research, Chinese Academy of Sciences, Shenyang 110016, China; gxcai@imr.ac.cn (G.C.); jmli@imr.ac.cn (J.L.); 3 School of Materials Science and Engineering, University of Science and Technology of China, Shenyang 110016, China; klliu22s@imr.ac.cn

**Keywords:** resistance spot weld, ultrasonic testing, non-destructive evaluation, mechanical property, spiral C-scan

## Abstract

In order to achieve the non-destructive testing and quality evaluation of stainless-steel resistance spot welding (RSW) joints, a portable ultrasonic spiral C-scan testing instrument was developed based on the principle of ultrasonic pulse reflection. A mathematical model for the quality evaluation of RSW joints was established, and the centroid of the ultrasonic C-scan image in the nugget zone of the RSW was determined based on the principle of static moment. The longest and shortest axes passing through the centroid in the image were extracted, and the ratio of the longest axis to the shortest axis (RLS) factor and the average of axis (AOA) factor were calculated, respectively, to evaluate the quality of the joint. To study the effectiveness of the detection results, tensile tests, and stereo analysis were conducted on the solder joints after sampling. The results indicate that this detection method can realize online detection and significantly improve the detection efficiency; the detection value of internal defect size is close to the true value with an error of 0.1 mm; the combination of RLS and AOA factors can be used to evaluate the mechanical properties of RSW joints. This technology can be used to solve the NDT, evaluate problems of RSW joints, and realize engineering applications.

## 1. Introduction

With the increasing progress of urbanization, the establishment of a modern rail transit system is the main way to solve urban transportation problems [1]. Among various types of urban rail vehicles, stainless-steel bodies have become the mainstream lightweight body of rail transit at home and abroad due to their clear advantages, such as simple manufacturing process, low operating costs, corrosion resistance, and long service life [2].

Stainless-steel vehicle bodies generally use thin sheets of 0.6–4 mm thickness. Due to the high electrical resistivity and low thermal conductivity of stainless-steel, welding deformation is prone to occur in the welding process, resulting in significant residual stress in the welding area [3]. Therefore, RSW, as a welding technique with low heat input, has become the preferred welding method for manufacturing stainless-steel urban rail vehicle bodies [4].

Generally, each stainless-steel vehicle body contains tens of thousands of welding points, and the quality of RSW joints directly affects the performance of the entire body. In the process of the increasing pursuit of high-speed rail vehicles, people also put forward more strict and higher standards for the safety and stability of the vehicle body. In the process of spot welding, the formation and growth of nuggets are both in a closed state and cannot be observed directly. At the same time, due to the extremely short time of nugget formation, the spot-welding process is prone to producing welding defects such as spatter, faulty welding, and Shrinkage cavity [5]. Therefore, non-destructive testing and the evaluation of RSW joints are crucial to ensure the service safety of rail transits. In order to achieve the non-destructive testing and evaluation of the quality of RSW joints, multiple research projects have been conducted at home and abroad, the main testing methods for which include online monitoring based on welding parameters, eddy current thermal imaging testing, and ultrasonic testing [6].

Online monitoring based on welding parameters refers to the prediction of spot-welding quality by monitoring the dynamic changes in process parameters such as welding electrode voltage and welding current. Especially with the rapid development of machine learning, the combination of deep learning and welding parameter monitoring has become mainstream in RSW quality prediction [7,8,9]. For example, Wan et al. [10], in order to achieve the reliable prediction of weld quality, used two neural network algorithms to predict the diameter of weld nuggets in spot welding joints by monitoring parameters such as welding electrode voltage and the welding current of TC2 titanium alloy RSW joints. Wen et al. [11] used regression analysis models and backpropagation neural network algorithms to identify spatter defects in RSW joints by dynamically monitoring electrode voltage signals and the rate of change in dynamic resistance. However, online monitoring is often applied to the welding process of RSW joints, so it is difficult to achieve in-service quality evaluation. Eddy current testing is based on the principle of electromagnetic induction; it uses sensors to pick up the signal changes in eddy currents in the workpiece to determine the size of defects. However, the eddy currents have a skin effect, so eddy current testing can only be used to detect surface and subsurface defects [12]. The emerging eddy current thermal imaging technology combines eddy current detection and infrared detection technology, uses the induced current to generate Joule heat in the workpiece, and collects temperature changes on the workpiece surface through an infrared thermal imager to identify the size of nugget and defects [13]. A. Taram et al. [14,15], on the one hand, used infrared cameras with a spatial resolution of 40 μM/pixel to monitor the heat conduction process in the joint, and better identify the size of the nugget based on the signal analysis and processing technology of Fourier transform. By comparing with the results of the destructive test, the measurement error of the nugget diameter was less than 1 mm. On the other hand, eddy current thermal imaging technology is applied to RSW crack detection. The phase information of the defect after filtering can be extracted by taking the changing image sequence, and the obtained crack defects are compared with the measurement results of the optical focusing microscope, both of which have good consistency. In engineering applications, RSW joints often have a large number and a wide distribution range, and the efficiency of the above methods is low, while the detection accuracy needs to be improved.

Researchers have also attempted to use the conventional ultrasonic C-scan to test RSW joints. For example, Jing Liu et al. [16,17] used a 15 MHz probe to study SUS304 stainless-steel RSW joints and revealed the propagation process of ultrasonic waves in typical RSW joints through a finite element simulation. Based on the BP neural network algorithm, they intelligently classified failure, stick welding, gas pore defects, and good joints, achieving a series of research results. However, the efficiency of conventional ultrasonic C-scan testing is relatively low. Therefore, in recent years, phased array ultrasonic testing technology has gradually been applied to measure the size of nuggets. This technology applies different time delay rules (focusing rules) to different units of the transducer array when transmitting or receiving ultrasonic reflection, achieving the movement, deflection, and focusing of the ultrasonic beam to improve detection efficiency [18]. For example, Bin Wang et al. used the Zetec Dynaray Lite-phased array ultrasonic flaw detector to manually inspect the DP590 dual-phase steel double-layer plate RSW joint specimens [19]. The relative error between the phased array ultrasonic testing and metallographic testing results was less than 5%. However, the electronic focusing rule of phased array results in certain blind spots during detection. When the upper plate of the joint is less than 1 mm, this technology is difficult to use to achieve the high-precision detection of the size of the nugget.

In summary, there is an urgent need for a reliable and efficient non-destructive testing method to detect and evaluate RSW joints. In this study, based on the structural characteristics of RSW joints, spiral scanning was used instead of conventional x-y scan. The ratio of the longest axis to the shortest axis (*RLS*) evaluation factor and the average of the axis (*AOA*) evaluation factor model are proposed for the irregular nugget zone to lay the foundation for achieving engineering applications in online quality inspection and the evaluation of RSW joints.

## 2. Principles of Ultrasonic Spiral C-Scan Test System

**Figure 1:**
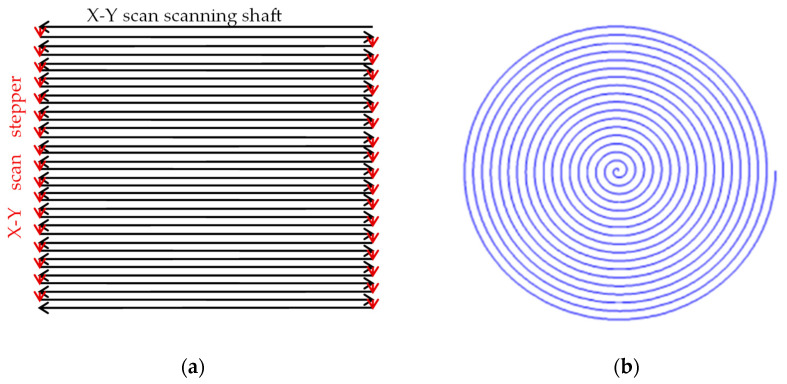
Comparison between X-Y scan and spiral scan. (**a**) X-Y scan; (**b**) spiral scan.

The conventional ultrasonic C-scan detection scanning method is the X-Y scan, as shown in Figure 1a, which is divided into a scanning axis and a stepper axis [20,21]. During the scanning process, frequent start–stop control of the stepper shaft motor is required, which not only causes mechanical impact but also seriously affects the detection speed.

Fast spiral scanning technology is proposed, which can reduce mechanical impact and significantly improve detection speed without the frequent startup and shutdown of the stepper shaft motor under the same scanning resolution. Taking the stepping accuracy of the stepper axis as an example of 0.1 mm, if the stepping length is 10 mm, about 100 s of time can be saved for each detection. When the step accuracy is smaller, and the step length is larger, more time will be saved.

**Figure 2:**
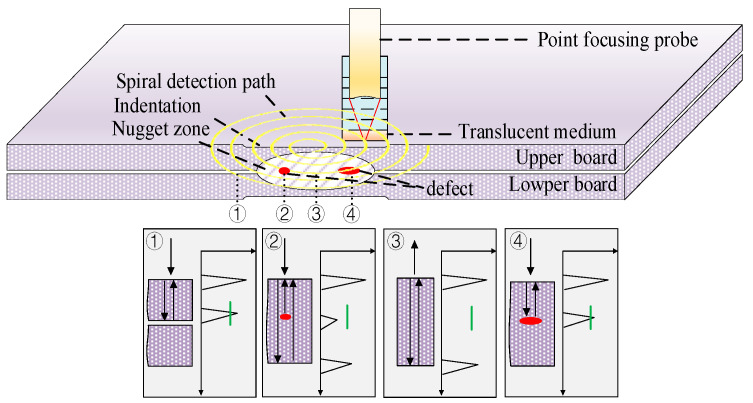
Principle of spiral c-scan ultrasonic detection.

As shown in Figure 2, during detection, the 25 MHz point focusing probe is focused on the surface of the upper plate first, which serves as both the transmitting probe and the receiving probe. The ultrasonic waves emitted by the probe propagate in a straight line in the tested workpiece after being coupled with water and a sound-translucent medium. After turning on the button, the rotating detection sensor connects the probe for spiral scanning, and the CPU processes the ultrasonic detection data in real-time. When the probe is located outside the nugget zone, an ultrasonic wave propagates to the bottom of the upper plate and reflects, forming a reflected echo (the bottom wave of the upper board) until it is received by the probe, as shown in Figure 2 ①. When there are small defects in the nugget zone, ultrasonic waves are reflected and refracted by the defect. The reflected ultrasonic wave is received by the probe, forming the defect echo. The refracted ultrasonic wave continues to propagate forward until it meets the bottom surface of the lower, forming a bottom wave (the bottom wave of the lower board), as shown in Figure 2 ②. When the probe is located in the nugget zone, and there are no defects inside, the ultrasonic wave propagates to the bottom surface of the lower board and reflects, forming the reflected echo that is received by the probe, as shown in Figure 2 ③. If there is a significant defect in the nugget zone, the ultrasonic wave reflects at the defect location, blocking the propagation path, and no bottom wave is formed, as shown in Figure 2 ④. Finally, the ultrasonic signals of different amplitudes inside the gate are extracted by setting the gate position, thereby achieving the ultrasonic C-scan imaging of the nugget zone.

**Figure 3:**
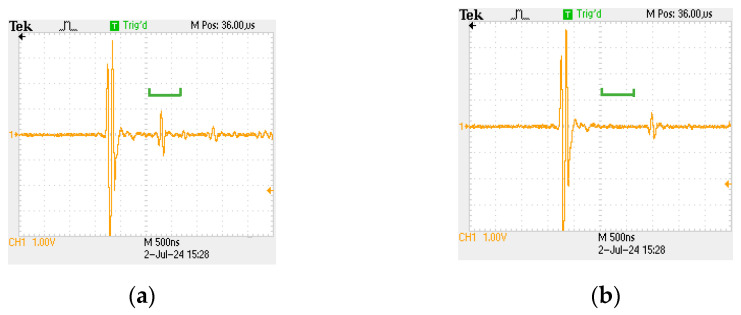
Ultrasonic reception signal. (**a**) Ultrasonic signal of the non-nugget zone; (**b**) ultrasonic signal of the nugget zone.

The system uses a negative pulse with an amplitude of 250 V and pulse width of 50 ns as the excitation signal, and the typical ultrasonic signal corresponding to the position of the non-nugget zone is shown in Figure 3a. The nugget zone is shown in Figure 3b. By calculating the thickness of the plate, the time for ultrasonic waves to propagate to the bottom of the upper board is calculated. The green gate position is set according to the time, and the amplitude of ultrasonic signals at different scanning positions inside the gate is extracted. Finally, a color gradient diagram of the joint is formed, which is the morphology of the nugget seen.

## 3. Structure of Ultrasonic Spiral C-Scan Test System and Data Processing of Test Results

### 3.1. Structure of Ultrasonic Spiral C-Scan Test System

**Figure 4:**
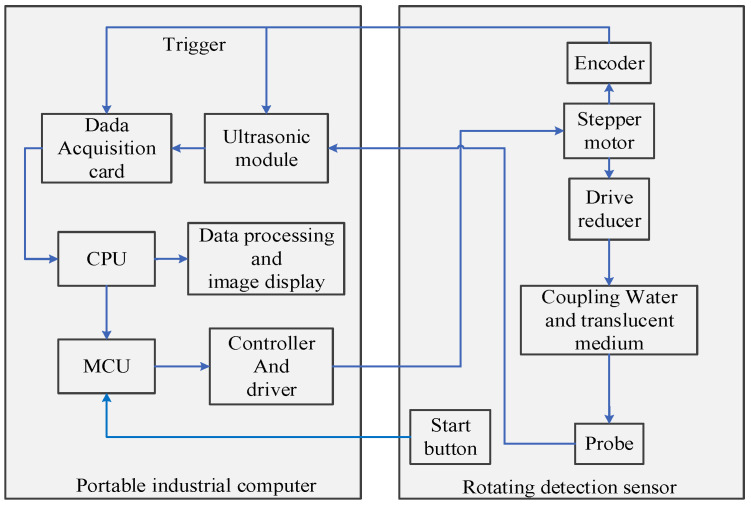
Basic composition of the instrument.

The ultrasonic spiral C-scan detection instrument includes two parts: a portable industrial host and a rotating detection sensor. The portable industrial host consists of an ultrasonic pulse transceiver, a data acquisition card, a controller, and a driver. The ultrasonic pulse transceiver is responsible for exciting the probe to emit ultrasonic waves and for amplifying and filtering the received ultrasonic echo signals. The data acquisition card is responsible for collecting the ultrasonic signals processed via the ultrasonic pulse transceiver and uploading them to the CPU; the controller and the driver control the stepper motor to scan the specified number of turns and adjust the encoder trigger signal. The instrument composition is shown in Figure 4.

**Figure 5:**
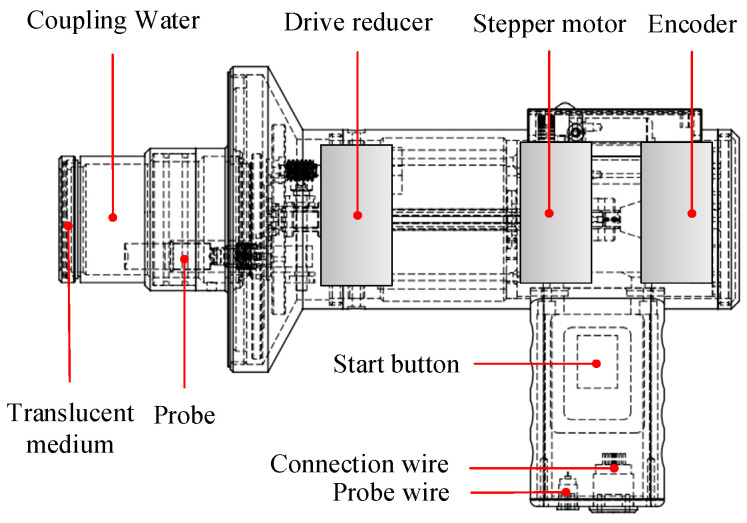
Composition of high-precision rotation detection sensor.

The high-precision rotation detection sensor mainly consists of the stepper motor, drive reducer, encoder, start button, coupling water, translucent medium, and probe. The upper computer software controls the operating parameters of the stepper motor and drives the detection probe to move along the progressive spiral line in the predetermined plane through the mechanical drive module. The encoder is matched with the precision radial stepper scanning sensor so that the scanning precision of the probe can reach 0.1 mm and the maximum scanning range can reach φ 20 mm, as shown in Figure 5. According to the AWS D8.7M: 2005 standard [22], when the plate thickness is 5 mm, it is required that the nugget diameter is greater than 9 mm. In the EN15085-3:2007 standard [23], when the plate thickness is 3 mm, the nugget diameter is required to be greater than 8.5 mm. Therefore, the scanning range of the high-precision rotation detection device is sufficient to cover the size of RSW joints in the fields of aerospace, rail transit, and automotive fields.

### 3.2. Data Processing of Test Results

The original basis for the quantitative evaluation obtains the morphology and size of weld nuggets through ultrasonic C-scan imaging. Domestic and foreign scholars have proposed different evaluation factors to scientifically and reasonably evaluate the quality of RSW joints. At present, the main evaluation factors are the nugget diameter, indentation depth, weld aspect ratio (*WAR*), defect size, and penetration rate. Defects are often inside the nugget zone, and if the defect size is not large, it has a smaller impact on the tensile strength of the joint. The penetration rate and indentation depth are often positively correlated with the melt core diameter [24]. Therefore, nugget diameter values and WAR are the two key evaluation parameters. In addition, attention should also be paid to the size of defects and their location in the nugget zone.

**Figure 6:**
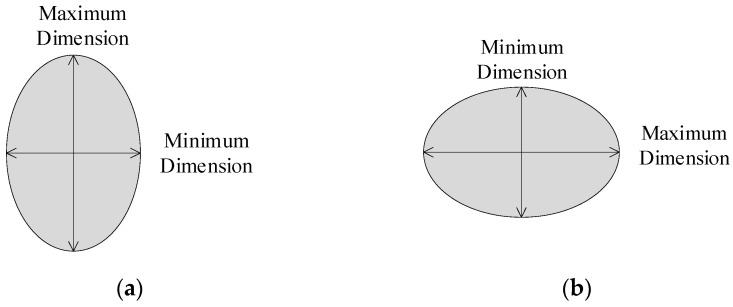
Measurement methods for nugget diameter under ideal nugget morphology. (**a**) Minimum size in transverse; (**b**) minimum size in longitudinal.

The AWS D8.7M: 2005 standard [22] specifies that the diameter of the nugget is half that of the sum of the maximum longitudinal (or transverse) size and the minimum transverse (or longitudinal) size, and the *WAR* is the ratio of the maximum longitudinal (or transverse) size compared to the minimum transverse (or longitudinal) size, as shown in Figure 6.

The diameter *D* of the nugget in the joint should be no less than four times the arithmetic square root of the measured plate thickness; the formula is as follows:

(1) $$D\geq 4\sqrt{t}$$

In the formula, *D* represents the diameter of the nugget, *t* represents the thickness of the test plate, and the *WAR* is no greater than two. The formula is as follows:

(2) $$WAR=\frac{Maximum\;Dimension}{Minimum\;Dimension}\leq 2$$

**Figure 7:**
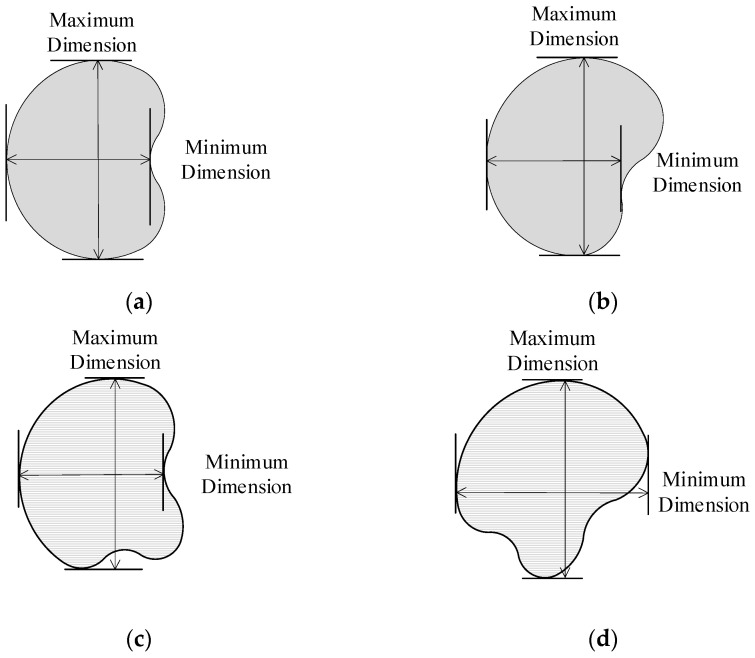
Morphology of nuggets at different angles of the same joint. (**a**,**b**) the same morphology of the nugget at different angles; (**c**,**d**) the same morphology of the nugget at different angles.

The morphology of the nuggets in Figure 6 is centrally symmetrical and an ideal morphology, with the center overlapping with the centroid. It is convenient to measure the maximum longitudinal (or transverse) size and minimum transverse (or longitudinal) size of the nuggets to determine *D* and *WAR*. However, due to the electrode wear and welding of process parameters in engineering, the morphology of the nuggets in the joint is not always the ideal morphology, as shown in Figure 6, such as a round-like or oval-like shape, and the center does not coincide with the centroid. If formulas 1 and 2 are still used for evaluation, it can lead to the inconsistent *D* and *WAR* of the nuggets in the same joint at different angles, as shown in Figure 7a–d, which is clearly not accurate. To solve the problem of difficulty in accurately measuring the size of nuggets with round-like or oval-like shapes, the *AOA* factor and the *RLS* factor are proposed to evaluate the quality of the joint.

**Figure 8:**
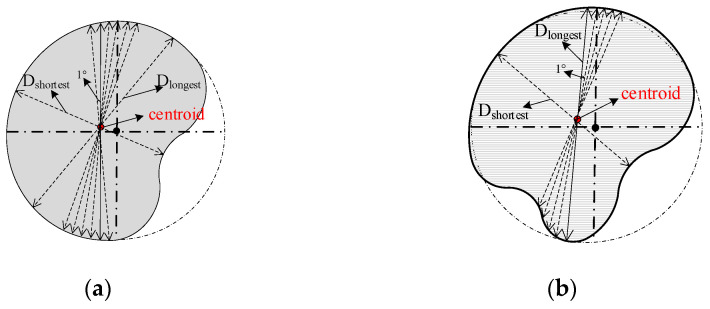
Mathematical model of *AOA* and *RLS* evaluation factors. (**a**) Example 1 of irregular morphology. (**b**) Example 2 of irregular morphology.

Based on the principle of static moment, the mathematical models of *AOA* and *RLS* factors were established to evaluate the quality of RSW joints. The specific implementation was to determine the centroid of irregular morphology of the nugget through the double integration algorithm, extract the length of 180 axis lines passing through this centroid with an interval of 1° between each axis line, and calculate the *AOA* evaluation factor and *RLS* evaluation factor, as shown in Figure 8.

Assuming that the irregular nugget morphology area in Figure 8 is a planar thin slice, the area in the *Oxy* plane is *σ*, mass is *M*, and density is *ρ*(*x*,*y*). So, the mass *M* is as follows:

(3) $$M=\iint_{D}\rho \left(xy\right)\mathrm{d}\sigma$$

Its centroid coordinates are ($\bar{x},\bar{y}$) as follows:

(4) $$\bar{x}=\frac{1}{M}\iint_{D}x\rho \left(xy\right)\mathrm{d}\sigma$$

(5) $$\bar{y}=\frac{1}{M}\iint_{D}y\rho \left(xy\right)\mathrm{d}\sigma$$

When *ρ*(*x*,*y*) is constant, the irregular region is uniformly distributed; then, the *M* = *ρσ*, coordinates ($\bar{x},\bar{y}$) are the centroid coordinates of the irregular plane region, and the centroid coordinates of the irregular two-dimensional image are derived as follows:

(6) $$\bar{x}=\frac{1}{\sigma }\iint_{D}x\mathrm{d}\sigma$$

(7) $$\bar{y}=\frac{1}{\sigma }\iint_{D}y\mathrm{d}\sigma$$

Considering the fact that the stress of the RSW joint in service is not simply the shear force parallel to the contact surface, but there are also the effects of the normal tensile force perpendicular to the contact surface, bending moment parallel to the contact surface, and torque perpendicular to the contact surface, the axis passing through the centroid can divide the irregular nugget morphology into two equal parts, and the axis in each direction can better represent the service performance of the joint in all directions. Therefore, the average length of the axis line in each direction of the nugget zone is taken instead of the nugget diameter to evaluate the quality of the solder joint; the formula is as follows:

(8) $$AOA=\frac{\sum_{1}^{180}D\;i}{180}\left(i=1\cdots \cdots 180\right)$$

From Figure 8, it can be seen that the centroid of the irregular region is clearly inconsistent with the center position of its circle, which is caused by the irregular morphology of the nuggets. When the morphology of the nugget in the joint is round or oval, the centroid of the nugget morphology should coincide with the center position of the circle in which it is located. Therefore, the *WAR* of the nugget can be characterized by the ratio of the longest axis line to the shortest axis line of the centroid in the morphology of the nugget, which is expressed as follows:

(9) $$RLS=\frac{D_{longest}}{D_{shortest}}\;$$

**Figure 9:**
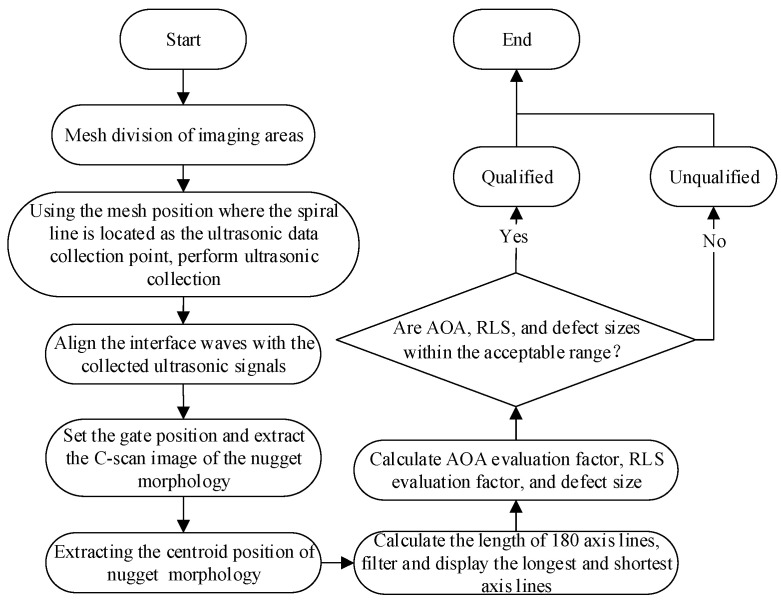
Software flow chart.

In the formula, $\;D_{longest}$ is the longest axis of the irregular nugget morphology $\;and{\;D}_{shortest}$ is the shortest axis of the irregular nugget morphology. Therefore, the combination of *AOA* and *RLS* can be used to evaluate the quality of the welded joints, which not only reflects the size and *WAR* of the weld nuggets in RSW joints but also avoids the problem of unreasonable evaluation results caused by the irregular morphology of the weld nuggets and has engineering significance. The upper computer software flow of the instrument is shown in Figure 9.

## 4. Experiments of Ultrasonic Spiral C-Scan Tests and Tensile Shear Tests

### 4.1. Specimen Preparation

**Figure 10:**
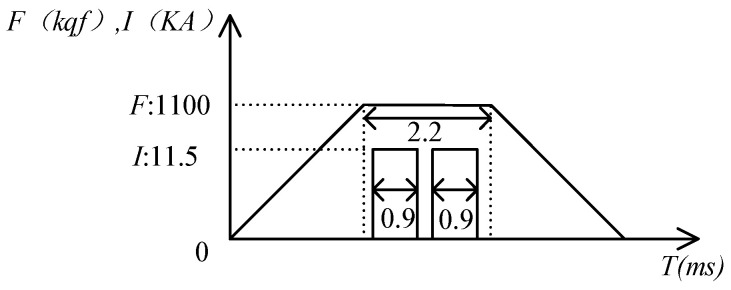
The welding process.

To simulate the RSW joints at the bottom frame of a stainless-steel train body, the Panasonic YR-500 resistance spot welding machine was used to prepare the RSW joints using the double-sided single spot-welding process with the C-shaped clamp with an electrode diameter of 23 mm. The welding process is shown in Figure 10.

The base material is machined and strengthened SUS301L-HT austenitic stainless-steel, with plate thickness combinations of 4 mm + 4 mm and 2 mm + 2 mm, is used. The chemical composition is shown in Table 1.

### 4.2. Experimental Ultrasonic Spiral C-Scan Tests and Tensile Shear Tests

Using the ultrasonic spiral C-scan detection instrument, 20 joints of double-layer plates welded under the above process were subjected to ultrasonic testing. The morphology of the melt was extracted using ultrasonic C-scan images, and the *AOA* evaluation factor, *RLS* evaluation factor, and defect size were calculated.

**Figure 11:**
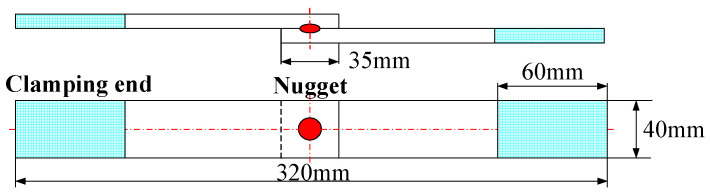
Dimensions of tensile shear specimen.

To verify the influence of the *AOA* evaluation factor and *RLS* evaluation factor on the service performance of joints, 16 joints were made into tension shear specimens according to the dimensions shown in Figure 11 to simulate the actual stress situation during the operation. The test referred to the JIS Z3136:1999 standard [25], and the RSW joints were subjected to room temperature tension using an electronic tensile testing machine model of Germany, Ruhr Industrial Zone, Zwick Z050, with a maximum load of 100 kN.

### 4.3. Result Analysis

Before using the instrument, sensitivity calibration is required, as described. Place the rotation detection sensor on the joint to be tested, adjust the bottom wave amplitude of the upper board of the joint to 80% of the full screen of the instrument, and maintain this detection setting before conducting actual testing.

#### 4.3.1. Verification of RSW Nugget Morphologies Obtained by Ultrasonic Spiral C-Scan

To verify the effectiveness and reliability of the detection results, the four joints are chiseled with destructive means, and the actual nugget morphology and internal defect size of the joints are compared with the ultrasonic detection results.

**Figure 12:**
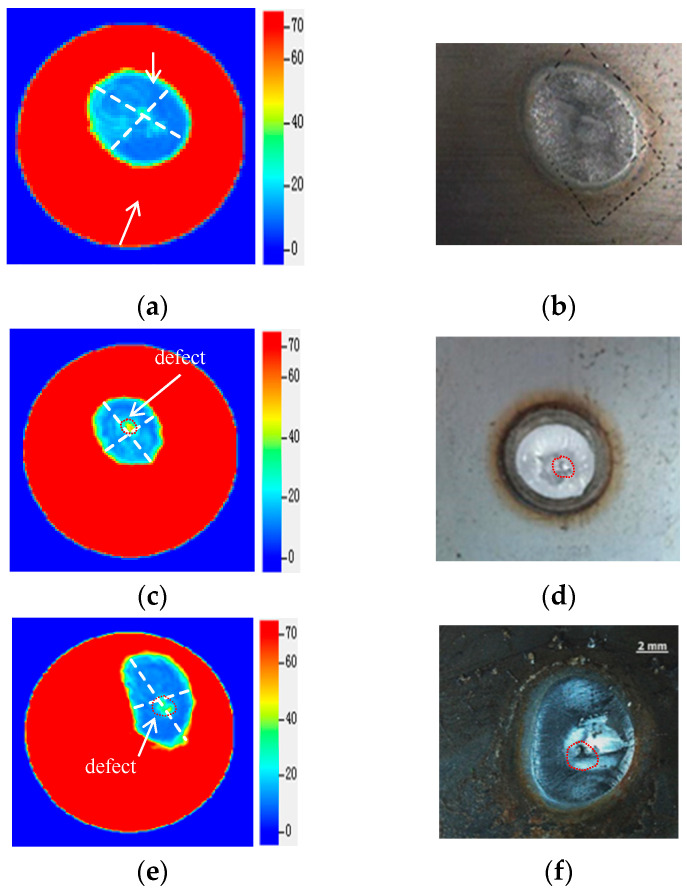

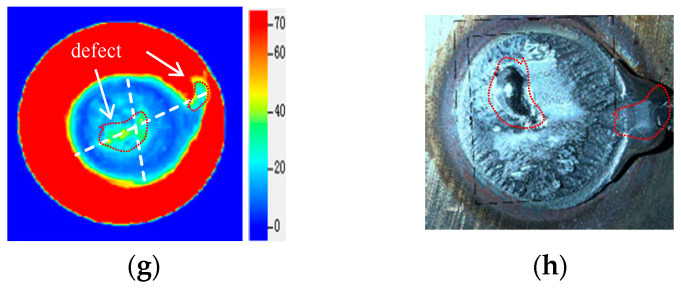
Comparison of morphological consistency. (**a**) Detected morphology of S−1 joint; (**b**) actual morphology of S−1 joint; (**c**) detected morphology of S−2 joint; (**d**) actual morphology of S−2 joint; (**e**) detected morphology of S−3 joint; (**f**) actual morphology of S−3 joint; (**g**) detected morphology of S−4 joint; and (**h**) actual morphology of S−4 joint.

From Figure 12, it can be seen that the ultrasonic C-scan results of the four joints are consistent with the actual tensile and shear fracture morphology. No defects were found in sample S-1 through ultrasonic C-scan imaging and the stereo measurement. Samples S-2, S-3, and S-4 all had a shrinkage cavity. In addition, sample S-4 had interplate splashing, and the detection error of defect size was less than 0.1 mm. As shown in Table 2.

From the comparison results, it can be seen that the developed ultrasonic spiral C-scan detection instrument had good consistency in detecting the types and sizes of the defects in the RSW joints, and the morphology and size of the defects could be visually and accurately displayed through instrument imaging.

#### 4.3.2. The Influence of Evaluation Factors on Mechanical Properties

**Figure 13:**
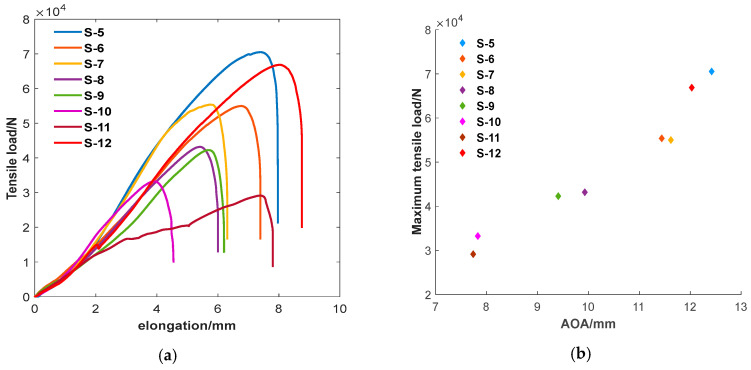
The relationship between *AOA* and mechanical properties. (**a**) Curves of tensile tests on specimens. (**b**) maximum tensile load of each specimen as a function of its *AOA* value.

To verify the influence of the *AOA* evaluation factor on the service performance of joints, eight joint specimens with good roundness and different *AOA* values were selected using ultrasonic spiral C-scan detection technology for room temperature tensile testing. The influence of the *AOA* evaluation factor on the mechanical properties of the joint was analyzed, as shown in Figure 13.

From Figure 13, it can be seen that there is a good positive correlation between the *AOA* evaluation factor and the maximum tensile shear force. If the Pearson correlation coefficient is calculated according to Formula (10), the correlation coefficient between the *AOA* evaluation factor and the maximum tensile shear force is 0.9717. Overall, as the *AOA* increases, the maximum tensile shear force also increases.

(10) $$r(X,Y)=\frac{Cov(X,Y)}{\sqrt{Var[X]Var[Y]}}\;\;\;\;\;\;\;\;\;$$

*r* (*X*, *Y*) represents the correlation coefficient, *Cov* (*X*, *Y*) is the covariance between *X* and *Y*, Var [*X*] is the variance of *X*, and Var [*Y*] is the variance of *Y*.

To verify the influence of the *RLS* evaluation factor on the service performance of joints, eight joints were selected and divided into four groups according to their *AOA* and *RLS* values measured by the ultrasonic spiral C-scan method. The eight joint specimens were selected so that their *AOA* and *RLS* values had the features for each of the four groups; the *AOA* values of the two joints were nearly equal to each other, but the *RLS* values were different from each other, and all the *RLS* values of the specimens A-1, B-1, C-1, and D-1 approached 1.00. The *RLS* value of each joint of the four groups of specimens measured by ultrasonic testing is listed in the second line of Table 3. Room temperature tensile tests on four 4 groups of joint specimens were conducted, and the obtained tensile shear strength of each joint was listed in the third line of Table 3. The tensile test curves and the variation in tensile shear strength of each joint with the *RLS* value of the joint are shown in Figure 13.

Figure 13 shows that there is a positive correlation between the *AOA* evaluation factor and the maximum tensile shear load, i.e., the strength of the joint. The *AOA* value is a measure of the size of the joint area. It is the area of the joint that bears the tensile shear force on the joint. Because of the above-mentioned mechanism, the *AOA* value has a great influence on the strength of the joint; consequently, it is the primary factor that controls the strength of the joint.

**Figure 14:**
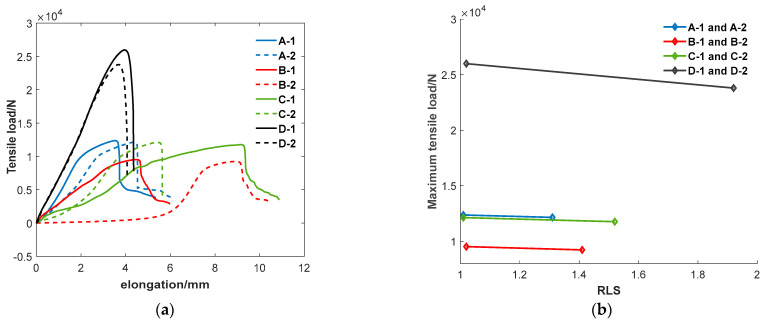
The relationship between *RLS* and mechanical properties. (**a**) Curves of tensile tests on specimens. (**b**) maximum tensile load of each specimen as a function of its *RLS* value.

Figure 14 demonstrates that there is a negative correlation between the *RLS* evaluation factor and the strength of the *RSW* joint. The *RLS* value is a measure of the shape of the joint area. Normally, the area of a plane figure depends on the *RLS* value when the *AOA* value is determined. The dependence is that the larger the *RLS* value, the smaller the area. Taking a rectangle or an ellipse as an example, the above-mentioned dependence is clear. Dependence is the reason why the strength of a joint is in negative correlation with the *RLS* value of the joint. Figure 14 shows that the strength of a RSW joint reduces slowly with the increase in the *RLS* value; therefore, the *RLS* value is a weak and secondary influence factor on the strength of the RSW joint compared with the *AOA* value. However, the correlation between *RLS* and the strength of the joint is complex; the influence of the *RLS* on the strength possibly depends on the angle of the longest axis with respect to the direction of the tensile force applied to the RSW joint. In order to know the correlation between *RLS* and the strength of the RSW joint in detail, more experiments and further analysis must be carried out. Due to the large amount of data [26], in the next research plan, deep learning techniques also need to be integrated.

## 5. Conclusions

The thin board structures, such as vehicle body shells, are extensively joined using spot welding. Ultrasonic C-scan imaging is the most important method for inspecting the weld points, as it can display the size, shape, and defects of the nugget zone.

This paper proposes and develops an ultrasonic spiral C-scan imaging technique and equipment. In comparison with traditional linear reciprocating scanning, the advantage is that it avoids the frequent start and stop of the motor and the mechanical impact. Meanwhile, under the same resolution conditions, the scanning speed is increased by three times.

The strength of the welding points has a significant impact on the quality and safety of the thin board structures joined by spot welding. This paper explores the non-destructive evaluation of weld point strength using ultrasonic C-scan. Therefore, not only was the ultrasonic C-scan imaging of the weld points conducted but tensile shear strength was also tested on several weld point specimens. The results of the analysis of the nugget zone image characteristics and the tensile shear strength of the specimens indicate that two parameters, the *AOA* of the average axis and the ratio *RLS* of the longest and shortest axis, are related to the shear strength of the weld points. *AOA* has a strong positive correlation with the shear strength, while *RLS* has a weak negative correlation with the shear strength. The above research lays the foundation for the further development of accurate and reliable ultrasonic non-destructive evaluation methods for weld point strength. Due to the complexity of the weld point strength issue, further research, experiments, and analysis are necessary.

## Figures and Tables

**Table 1:** Chemical composition of base metal.

C	Si	Mn	P	S	Ni	Cr	N	Fe
0.03	1.00	2.00	0.045	0.03	7.10	17.70	0.2	Bal

**Table 2:** Comparison between detection results and actual defect sizes(mm).

Sample Number	S-1	S-2	S-3	S-4
Detected defect size	0	1.18	1.36	2.05
Actual defect size	0	1.25	1.40	1.94
Error (Unit in mm)	0	−0.07	−0.04	0.09
Error (Unit in %)	0%	−5.9%	2.9%	4.3%

**Table 3:** The *RLS* value and joint strength of each of the 8 specimens.

Sample Number	A-1	A-2	B-1	B-2	C-1	C-2	D-1	D-2
*RLS* value	1.01	1.31	1.02	1.41	1.01	1.52	1.02	1.92
Maximum tensile load (kN)	12.37	12.15	9.52	9.23	12.14	11.77	26.00	23.80

## Data Availability

Data are contained within the article. The data presented in this study are available in Section 4.3.
